# The Belgrave Hospital for Children

**Published:** 1903-07-25

**Authors:** 


### HOSPITAL ADMINISTRATION.

### CONSTRUCTION AND ECONOMICS.

### THE BELGRAVE HOSPITAL FOR CHILDREN.

ON the 20th inst. H.R.H. Princess Henry of Battenberg visited the Belgrave Hospital for Children for the purpose of performing the opening ceremony. This hospital was formerly situated in Pimlico, but has been removed from thence and built on its present site in the Clapham Road amidst a large and far poorer population for whom there is no such accommodation. Some three years ago the foundation stone was laid by Princess Henry of Battenberg, and at the present time the centre, east, and part of the south wings, and out-patients' building and mortuary have already been built, comprising three wards with cots, babies' ward (35 cots in all), and offices for staff.

Large crowds assembled near the hospital some time before the ceremony took place; the roadway was gaily decorated with flags, and red baize had been laid down over the steps. The room in which the opening ceremony took place, the amputation receiving-room, proved quite inadequate to hold the numbers of spectators who were desirous of obtaining entrance, and when all standing room had been occupied, there yet remained a good number who were obliged to remain in the rooms adjoining. Her Royal Highness, who was attended by Lord William Cecil and Lady Windsor, was received on her arrival by Lord Windsor, Chairman of the hospital, and members of the reception committee, and was conducted over the hospital. The inspection occupied some little time and when it was concluded the Princess took her seat on the platform in the room where the guests were awaiting her, and a short service was conducted by the Revs. J. Darlington and R. Harris Lloyd.

Lord Windsor, in the course of a short speech alluded to the laying of the foundation stone by the Princess, and hoped that her Royal Highness would be pleased with the new building, which had been completed as far as the funds would allow. The sum of money spent upon the building up to now was £30,500 and £4,000 for the site. The hospital was about £10,000 in debt, and there was also a further sum of £5,000 lacking, making a total of £15,000 which was really required to complete the fittings of the present building. He ventured to hope that by the kindness of the supporters of the hospital and friends in general they would be enabled to pay this sum off before very long. An anonymous donor had offered £2,000 on condition that a like amount was contributed. They had already received £958 towards the first £1,000. The fittings of the rooms had been most carefully looked after by the committees into whose hands this duty had been entrusted. He could not omit to mention the very hard and successful work done by the Secretary, Mr. F. Stuart, during the past four years. The Ladies' Committee had raised £200 for cots and equipment, whilst two members of the committee had offered each to furnish one ward at a cost of £130. Lady Tate had generously given £1,000 for the endowment of a cot. With gifts such as these he hoped that within a very short time her Royal Highness would be able to see the hospital, fully equipped, doing the valuable work that all who were interested in it hoped it would be able to do in the midst of this populous district.

Princess Henry of Battenberg then in a clear voice said: "In the faith of Jesus Christ, I declare these buildings open; in the name of the Father, and of the Son, and of the Holy Ghost. Amen."

The opening ceremony having now been completed, her Royal Highness said: "I have much pleasure in consenting to one ward being called the 'Princess Beatrice' ward, and I would suggest that the other be called the 'Martineau' ward."

This last ward is named after Mr. J. Martineau, a member of the Committee of Management.

A vote of thanks to the Princess was then moved by the Mayor of Lambeth, who remarked that the district of Lambeth alone contained a population of 300,000. He announced that a further donation of £60 had been received, which assured them of receiving the first £1,000 promised conditionally. He thanked her Royal Highness very heartily for coming into their midst and performing the opening ceremony.

Sir F. W. Cook seconded the motion, which was carried by acclamation.

Tea was furnished to the guests, who availed themselves in large numbers of the opportunity of inspecting the, as yet tenantless, hospital.

The new hospital, which is entirely built of red brick has a frontage of 150 feet and a depth of 150 feet. The babies' ward on the first floor is a bright cheerful room charmingly decorated with coloured tiles representing nursery rhymes; the floor in this as in all the wards is of wood blocks, whilst in the passages it is of concrete. The skirting boards in all the rooms are well bevelled at the bottom so as to prevent any dust being harboured. The operating theatre is a well-lighted airy room, fitted up with all modern appliances. In an ante-chamber is the apparatus for regulating the temperature of the electric air current which, at the will of the surgeon, may be rendered hot or cold or any gradation necessary. The wards are all tiled half-way up. There is an outer iron staircase from the top stories.

To complete the whole hospital as planned, to give additional wards and make a total of 78 cots with all necessary offices for staff and equipment a sum of £25,000 is required.

